# The role of Apolipoprotein E epsilon4 in the association between psychosocial working conditions and dementia

**DOI:** 10.18632/aging.102843

**Published:** 2020-02-20

**Authors:** Kuan-Yu Pan, Weili Xu, Francesca Mangialasche, Giulia Grande, Laura Fratiglioni, Hui-Xin Wang

**Affiliations:** 1 Aging Research Center, Department of Neurobiology, Care Sciences and Society, Karolinska Institutet and Stockholm University, Stockholm 171 65, Sweden; 2 Division of Clinical Geriatrics, Center for Alzheimer Research, Department of Neurobiology, Care Sciences and Society, Karolinska Institutet, Solna 171 64, Sweden; 3 Stockholm Gerontology Research Center, Stockholm 113 30, Sweden; 4 Stress Research Institute, Stockholm University, Stockholm 114 19, Sweden

**Keywords:** psychosocial work environment, e4 *APOE*, dementia, cohort study

## Abstract

In this population-based prospective study, we examined the association of job demand-control combinations with dementia, and explored the roles of Apolipoprotein E epsilon4 (*APOE* ɛ4) and work duration in this association. A total of 2,579 dementia-free individuals aged 60+ years from Sweden were followed over 12 years. Dementia diagnosis was made by physicians. Lifelong occupational experience was collected, and job demands and control were assessed using a psychosocial job-exposure matrix. Data were analyzed using multivariate Cox proportional hazard models. During the follow-up, 282 people developed dementia. Passive jobs (low control/low demands) were related to a higher risk of dementia compared with active jobs (high control/high demands) among the younger-old (aged ≤72 years), but not among the older-old (aged ≥78 years). Among the younger-old, compared to those with no passive job experience, those with 11+ years in passive jobs had a higher dementia risk. The joint-effect analyses showed that *APOE* ɛ4 carriers with passive jobs had an even higher risk of dementia compared to *APOE* ɛ4 non-carriers with active jobs. These findings suggest that passive jobs are related to a higher dementia risk among the younger-old. *APOE* ɛ4 and long work duration may amplify the impact of passive jobs on dementia.

## INTRODUCTION

Dementia is a multifactorial disorder characterized by progressive deterioration in multiple cognitive domains severe enough to interfere with daily functioning [1]. Worldwide, around 50 million people are living with dementia, and the number is projected to multiply in the coming decades [2]. Genetic susceptibility and environmental factors (e.g., psychosocial and lifestyle factors), as well as their interaction over the lifespan, contribute to the pathological process and clinical expression of the disease [1].

Epidemiological studies have related psychosocial working conditions to late-life cognition and dementia, but the evidence is still limited. One of the most prominent models to evaluate psychosocial work environment is the job demand-control model [3]. This model is based on two components, job control and job demands, and subsequently generates demand-control combinations. Job control refers to the extent of autonomy to make decisions and utilize skills in conducting work tasks, whereas job demands represent psychological demands, mental workload, and time restrictions for completing the excessive amount of work. High strain (high demands/low control) refers to a stressful work scenario that may affect the brain through the stress response [4]. Passive jobs (low demands/low control), on the other hand, entail lack of motivation and mental stimulation, and may be detrimental for learning capacity and cognitive performance [3]. Both high strain and passive jobs have been associated with worse cognitive function in late life [5–7]. Studies focusing on dementia reported that low job control increased the risk of dementia [8–11], but no association between job demands and dementia was observed in one study [12]. In a previous study we reported the relationship of high strain and passive jobs to a higher risk of dementia using data from the Kungsholmen Project (KP) [10].

The Apolipoprotein E epsilon4 (*APOE* ɛ4), an important genetic risk factor for dementia, has been found to modify the association between environmental factors and dementia [13, 14]. However, the interaction between psychosocial working conditions and *APOE* ɛ4 to dementia has rarely been investigated. Recently, one study showed that low job control modified the association between *APOE* ɛ4 and dementia [11].

Moreover, when examining working conditions in relation to late-life cognitive function and dementia, it is essential to consider the duration of work throughout working life [15]. Studies on job demands and control and dementia have attempted to take into account work duration, either by assessing the longest-held job [9, 10] or by weighing each occupation in life against its duration [8]. Despite this, the impact of demand-control status, and its duration, on dementia risk has not been fully identified.

In the current study, we used a different study population that, compared to the KP cohort, not only has a larger sample and longer follow-up time, but also has more diversity with respect to age/birth cohort and the access to genetic information. We aimed to 1) examine the association between job demand-control status and dementia, 2) verify the working conditions-*APOE* ɛ4 interaction, and 3) investigate whether longer work duration amplifies the impact of detrimental working conditions on dementia.

## RESULTS

### Characteristics of the study population

Of the 2,579 participants at baseline (mean age 72.7±10.4), 1,602 (62%) were women. People who had worked in passive jobs were less educated and with lower early-life socioeconomic status (SES), and more likely to have heart diseases. Those with active jobs were younger, more likely to be men and engage in leisure activities, and had a higher baseline Mini-Mental State Examination (MMSE) score. No difference was observed in *APOE* allelic status across demand-control categories (Table 1). We observed a similar pattern when comparing characteristics of participants in the two age cohorts, the younger-old (aged ≤72 years) and the older-old (aged ≥78 years), separately (Supplementary Tables 1 and 2).

**Table 1:** Characteristics of study population by demand-control status of the longest-held job.

**Characteristics**	**Active**		**Low Strain**		**High Strain**		**Passive**	***p***
	n=1611		n= 421		n=292		n=255	
Age (years)	71 ± 10		75 ± 10*		74 ± 10*		77 ± 11*	<0.001
Female sex	915 (56.8)		316 (75.1)		199 (68.2)		172 (67.5)	<0.001
Education								
Elementary	107 (6.6)		84 (20.0)		61 (20.9)		125 (49.0)	<0.001
High school	725 (45.0)		276 (65.6)		172 (58.9)		113 (44.3)	
University	779 (48.4)		61 (14.5)		59 (20.2)		17 (6.7)	
Physical leisure activity								
Low	296 (19.8)		86 (23.3)		49 (19.4)		47 (22.4)	
Moderate	784 (52.6)		213 (57.7)		135 (53.3)		131 (62.4)	<0.001
High	411 (27.6)		70 (19.0)		69 (27.3)		32 (15.2)	
Mental leisure activity								
Low	490 (33.6)		168 (47.1)		109 (44.7)		114 (55.3)	
Moderate	554 (37.9)		131 (36.7)		88 (36.1)		69 (33.5)	<0.001
High	416 (28.5)		58 (16.2)		47 (19.2)		23 (11.2)	
Social leisure activity								
Low	874 (59.2)		243 (66.9)		142 (57.3)		138 (66.7)	
Moderate	371 (25.1)		80 (22.1)		68 (27.4)		42 (20.3)	<0.05
High	232 (15.7)		40 (11.0)		38 (15.3)		27 (13.0)	
Early-life SES								
Low	513 (33.2)		187 (47.1)		137 (49.5)		141 (58.8)	
Intermediate	506 (32.7)		109 (27.5)		69 (24.9)		62 (25.8)	<0.001
High	528 (34.1)		101 (25.4)		71 (25.6)		37 (15.4)	
Heart diseases	335 (20.8)		125 (29.7)		66 (22.6)		87 (34.1)	<0.001
*APOE* ɛ4 carriers	450 (29.3)		110 (28.1)		78 (28.5)		67 (28.4)	0.97
MMSE score	29.0 ± 1.3		28.4 ± 1.8*		28.6 ± 1.6*		27.9 ± 2.1*	<0.001

Data are presented as mean ± standard deviations or number (proportion %).

Heart diseases include atrial fibrillation, ischemic heart disease, heart failure, cardiac valve disease, bradycardias and conduction diseases.

Abbreviations: SES, socioeconomic status; *APOE* ɛ4, apolipoprotein E ɛ4; MMSE, Mini-Mental State Examination.

Missing data: Physical leisure activity=256, Mental leisure activity=312, Social leisure activity=284, Early-life SES=118, *APOE* ɛ4=141, MMSE=10.

* Pairwise mean comparison using Bonferroni correction: p<0.05 (reference group was active job).

Over the 12 years of follow-up (mean 8.8±3.7 years; 22,636 person-years), 282 participants developed dementia (151 Alzheimer’s type, 32 vascular, 41 mixed and 58 other types of dementia), of which 63 were younger-old and 219 were older-old. In both groups, incident dementia cases were less educated, more likely to carry *APOE* ɛ4, and had a lower baseline MMSE score. Among the younger-old, cases were older and more likely to have heart diseases (Supplementary Table 3).

### Psychosocial working conditions, *APOE ɛ4*, and dementia

In the entire study population, demand-control status of the longest-held job was not significantly related to dementia. After stratified by age, we found that passive jobs - compared with active jobs - were associated with an increased risk of dementia among the younger-old individuals, but not among the older-old (Table 2). When job demands and job control were treated as separate exposures, no statistically significant association of job control or job demands with dementia was observed (Supplementary Table 4).

**Table 2:** Hazard ratios (HRs) and 95% confidence intervals (CIs) of incident dementia associated with demand-control categories among the total sample and among two age cohorts.

**Job category**	**N**	**No. of cases**	**Model 1^a^**		**Model 2^b^**	
			**HR (95% CI)**	***p***	**HR (95% CI)**	***p***
**Overall**						
Active	1611	151	Ref.		Ref.	
Low strain	421	55	1.11 (0.80-1.53)	0.54	1.07 (0.77-1.50)	0.67
High strain	292	37	1.05 (0.72-1.53)	0.80	1.03 (0.70-1.53)	0.87
Passive	255	39	1.02 (0.69-1.49)	0.93	1.01 (0.68-1.49)	0.96
**Aged ≤72**						
Active	1064	40	Ref.		Ref.	
Low strain	196	10	0.85 (0.41-1.76)	0.66	0.77 (0.36-1.66)	0.50
High strain	158	4	0.40 (0.12-1.30)	0.13	0.39 (0.12-1.29)	0.12
Passive	98	9	2.36 (1.09-5.15)	<0.05	2.50 (1.10-5.67)	<0.05
**Aged ≥78**						
Active	547	111	Ref.		Ref.	
Low strain	225	45	1.11 (0.77-1.59)	0.59	1.07 (0.74-1.56)	0.72
High strain	134	33	1.23 (0.82-1.84)	0.31	1.16 (0.76-1.77)	0.50
Passive	157	30	0.92 (0.60-1.42)	0.71	0.90 (0.58-1.41)	0.65

^a^ Adjusted for age, sex, and education; ^b^ Adjusted for age, sex, education, heart diseases, leisure activity engagement, and early-life socioeconomic status.

**Figure 1:**
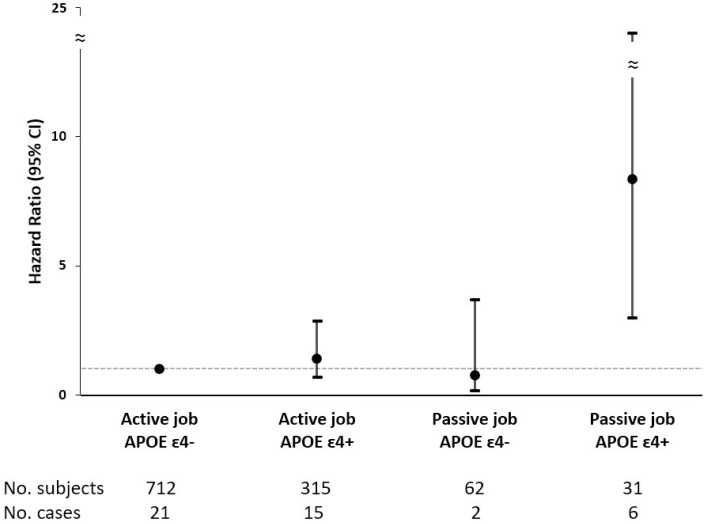
**Joint effect of demand-control status and *APOE* ɛ4 on incident dementia among adults aged ≤72 years.** Hazard ratios (HRs) and 95% confidence intervals (CIs) derived from Cox regression model adjusted for age, sex, education, heart diseases, leisure activity engagement, and early-life socioeconomic status. *APOE* ɛ4, apolipoprotein E ɛ4.

In addition, we detected a multiplicative interaction between passive jobs and *APOE* ɛ4 with respect to estimating dementia risk among the younger-old (p<0.05), but not among the older-old. No statistically significant interaction between working conditions and sex, education, or early-life SES was found. In the joint effect analyses among the younger-old, *APOE* ɛ4 carriers with passive jobs had a higher risk of dementia, as compared to *APOE* ɛ4 non-carriers with active jobs (HR 8.21, 95% CI 2.91-23.15) (Figure 1).

### Duration of passive jobs throughout working life and dementia

**Figure 2:**
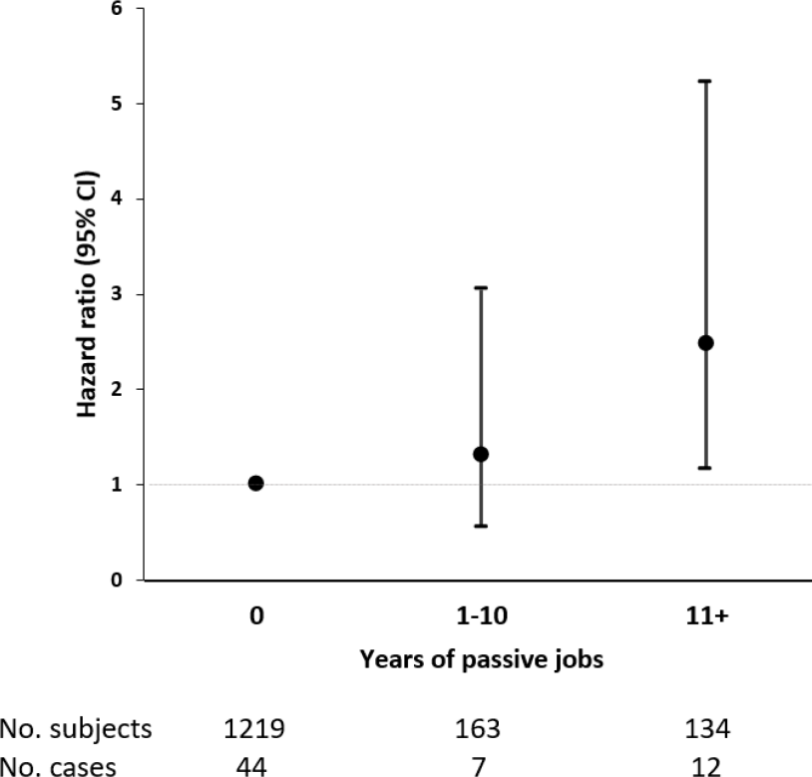
**Hazard ratios (HRs) and 95% confidence intervals (CIs) of incident dementia associated with duration of passive jobs among adults aged ≤72 years.** Cox regression model was adjusted for age, sex, education, heart diseases, leisure activity engagement, and early-life socioeconomic status.

Since passive job was the only demand-control category to be associated with dementia, we focused on this category in analyses regarding duration of work in relation to dementia. As in our study population the median duration spent in passive jobs was ten years, we created a three-category variable: zero, less than or equal to ten, and more than ten years. Following the same stratified analyses, among the younger-old, in comparison with people with no passive job at all during their entire working lives, those who had worked in passive jobs for more than ten years demonstrated an increased risk of dementia (HR 2.54, 95% CI 1.21-5.35) (Figure 2). No association between duration of passive jobs and dementia was found among the older-old (Supplementary Figure 1). All sensitivity analyses produced similar results to those from initial analyses.

## DISCUSSION

In this population-based cohort study, the Swedish National Study on Aging and Care-Kungsholmen (SNAC-K), we found that passive jobs were associated with a higher risk of dementia among the younger-old (aged ≤72 years), but not among the older-old (aged ≥78 years). Among the younger-old, there was an interaction between *APOE* ɛ4 and passive jobs in terms of dementia occurrence. Having long duration of more than ten years in passive jobs was related to a higher dementia risk.

While previous studies reported that low control [8, 9, 11] or low demands [8] were associated with an increased risk of dementia, none of them tested the relationship of job demands and control to dementia using categories in accordance with the demand-control model. Our finding that passive jobs, which include both low control and low demands, were related to an increased risk of dementia was also observed in our previous work using the KP population [10]. Passive jobs were also linked to poorer cognitive function in later life [5–7]. In the SNAC-K cohort we found that passive jobs were associated with accelerated cognitive decline [7].

One of the biological mechanisms that underlies the link of passive jobs to poor cognitive function and dementia may be related to limited cognitive reserve. The concept of cognitive reserve suggests that the brain tends to actively cope with brain changes by using preexisting neuronal processing approaches or by generating compensatory networks to maintain cognitive function [16]. Having a passive job infers a lack of motivation and mental stimulation at work, which in turn may fail to preserve neuronal activity or to provide compensatory networks necessary to maintain cognitive function during brain changes. In addition, having a passive job could also be perceived as a source of stress due to the lack of development and self-efficacy [17]. The hypersecretion of glucocorticoid hormone from stress response has been suggested to be detrimental to the brain and cognition [4].

In the current study, we found that passive jobs increased the risk of dementia due to *APOE* ɛ4. This is in line with the previous study showing that low control, a component of passive jobs, magnified the impact of *APOE* ɛ4 on dementia [11]. Similarly, an interaction was also observed between passive jobs and *APOE* ɛ4 among the younger-old participants in our previous work focusing on cognitive decline [7]. *APOE* ɛ4 has been associated with the accumulation of neurofibrillary tangles and neuritic plaques [18], and might entail an increased neuronal vulnerability to environmental factors. *APOE* ɛ4 carriers have been reported to be more susceptible to environmental risk factors for dementia, including smoking, heavy alcohol consumption, physical inactivity, and high intake of saturated fats [13]. On the other hand, it has been shown that education, job complexity, leisure activities, and social network may attenuate the genetic risk of dementia due to *APOE* ɛ4 [14], possibly by enhancing cognitive reserve and reducing stress levels. Similarly, in this study, active jobs appeared to nullify the detrimental effect of genetic predisposition, as the risk of dementia among *APOE* ɛ4 carriers with active jobs was similar to that of the non-*APOE* ɛ4 carriers with active jobs.

Interestingly, the results from SNAC-K and KP seemed to go in different directions. In the KP population comprising older adults aged 75+ years during 1987-1989, we found a relationship between high strain and passive jobs and dementia [10]. By contrast, no association was shown between these working conditions and dementia among the older-old individuals of the SNAC-K population (aged 78+ years during 2001-2004). The discrepancy between results from KP and SNAC-K may have several explanations. As previously noted, passive jobs indicate demotivation and insufficient mental stimulation at work and have been associated with low cognitive reserve [16], whereas education acts as a shield against dementia by inducing cognitive reserve [19]. As the educational level of the older-old with passive jobs in SNAC-K was higher than that in KP, the connection of passive jobs to dementia might have been attenuated by more education. On the other hand, high strain represents a stressful work scenario and may be linked to dementia through cardiovascular disease (CVD) [19, 20]. The structure of the Swedish labor market has been reformed since the 1960s [21], and there have been ongoing efforts from the Work Environment Authority to improve working conditions since the 1970s [22]. Therefore, working conditions in Sweden may have changed, or possibly improved, over the last decades. Moreover, there has been an improvement in the control of CVD and a reduction in vascular risk factors between the 1980s and early 2000s in the Swedish population [23, 24]. Thus, as a result of changes in the work environment and cardiovascular risk factors, the impact of high strain on dementia may have become weaker in the SNAC-K population, as compared with the KP population. Taken together, the discrepancy between findings from the KP (year 1987-1989) and SNAC-K (year 2001-2004) may imply potential cohort effects on the association between working conditions and dementia in the Swedish population over twenty years. Nevertheless, more studies from different disciplines, including medical and social sciences, are needed to confirm the cohort effects that we speculated here.

There are other possible explanations for the association between passive jobs and dementia being limited to the younger-old participants. There might be a limited time window beyond working life, during which previous exposure to psychosocial working conditions can be related to health outcomes in later life [15]. One study found that higher work stress, characterized by a constant rush, increased the risk of dementia among the younger-old (mean age 71 years), but not among the older-old (mean age 78 years) [25]. To our knowledge, the current study is the first to identify such a time window using the demand-control model. As the majority of participants retired at the age of 65, our results may suggest that working conditions are only related to dementia occurrence within approximately ten years after retirement. The other possible explanation for this result may be survival bias. Passive jobs have been associated with increased mortality [26], therefore survivors among the older-old with passive jobs might be more resilient and less likely to develop dementia. While we also identified dementia cases among deceased subjects, the survival issue may have been partially addressed here. In addition, the higher heterogeneity among older-old dementia cases could result from a more complex combination of risk factors and chronic conditions [27], which may dilute the effect of a single psychosocial factor on dementia.

The current study explored how long it takes for the cumulative effect of passive jobs to impact dementia. A threshold of ten years of work in passive jobs was detected. This supports the hypothesis that unfavorable working conditions may become harmful to health when the exposure is long-lasting or repeated [15]. Future studies concerning job demands and control and dementia should address the role of duration of exposure, while also confirming the cumulative exposure threshold that we observed here.

The strengths of this study include the prospective design with a large population-based sample, the comprehensive source for a clinical diagnosis of dementia, and the availability of genetic data. The lifelong occupational information enabled assessment of job demand and control from different chronological perspectives, including the longest-held job and total years spent in different working conditions across the life span. The individual predispositions (i.e., education and early-life SES) that might preselect participants into occupations were also considered. Additionally, by replicating our previous study using a cohort from the same region twenty years later, we demonstrated the potential cohort effect on the work-dementia association.

The following limitations should be considered. First and foremost, our results from the stratified analyses should be interpreted with caution considering the small number of subjects and cases in the strata, especially among the younger-old age cohorts and *APOE* ɛ4 carriers. Second, lifelong career information was self-reported retrospectively, which could introduce information bias. To address the issue of misclassification, sensitivity analyses were conducted using the most recent, rather than longest-held occupations. We also excluded participants with potential cognitive impairment or preclinical dementia, and the results remained similar. Third, we relied on a psychosocial job-exposure matrix to measure job demands and control. Despite the reported reliability and validity of this matrix [28], variabilities in individuals’ perception on working conditions or job characteristics within occupations were not taken into account. But this approach may reduce self-reporting bias. Fourth, the self-selected participation of the older-old individuals may have resulted in underestimating the association between working conditions and dementia among this age group. Finally, the higher proportion of people in active jobs, as well as the higher frequency of *APOE* ɛ4 of the SNAC-K sample compared to other Western populations [29], may have limited the generalizability of our findings.

In conclusion, our study shows that psychosocial working conditions, in terms of passive jobs, are related to a higher risk of dementia among the younger-old adults. *APOE* ɛ4 and long work duration seem to enhance the impact of such negative work scenario on dementia occurrence. Considering the sample size of younger-old individuals in our study, more studies are warranted to confirm the link of job demand-control status to dementia and the modifying effect of *APOE* ɛ4 allele.

## MATERIALS AND METHODS

### Study population

The study population was from the SNAC-K, an ongoing, population-based observational survey, comprised of a random sample of individuals aged 60 years and older living in Stockholm’s Kungsholmen district [30]. At baseline (2001 to 2004), subjects were grouped into eleven age cohorts, with an interval of six years between the younger age cohorts (60, 66, and 72 years old), and three years between the older cohorts (78 years and older). Follow-up examinations were carried out every six years for the younger age cohorts and every three years for the older age cohorts. Of the 4,590 individuals who were alive and eligible, 3,363 (73.3%) attended the baseline examination. Of these, we excluded prevalent dementia cases (n=310), those who were alive but refused to attend the follow-up examinations (n=368), and those with a developmental disorder (n=1) or with missing occupational information (n=105). Thus, there were 2,579 participants remaining in the current study, with follow-up data up to 2016. A flow chart of the study participation can be seen in Supplementary Figure 2.

All waves of the SNAC-K study complied with the ethical standards of the Helsinki Declaration and were approved by the Karolinska Institutet Ethics Committee and the Regional Ethics Review Board in Stockholm, Sweden. Written informed consent was obtained from all participants or their next of kin.

### Data collection

SNAC-K data collection followed a structured protocol at baseline and all follow-ups (available at http://www.snac-k.se/). Information on demographic factors (age, sex, and education), occupational experience, lifestyle factors, early-life SES, and current medical conditions and medication use were collected through interviews and clinical examinations by nurses and physicians. Peripheral blood samples were obtained from all participants. Global cognitive function was evaluated using the MMSE conducted by physicians.

### Assessment of psychosocial working conditions

Lifelong occupational information including employer, job title, task and time span of the most recent job and four longest-held jobs was collected at baseline [30]. Each occupation was issued a 3-digit code in accordance with the Nordic occupation classification. Levels of job control and demands in each job were assessed through a validated psychosocial job-exposure matrix [28], which was constructed based on data from the Swedish Work Environment Survey, for men and women separately. This matrix has been used in other studies on the association between demand-control conditions and a variety of outcomes, including CVD, diabetes, and dementia [10, 31, 32].

In the matrix, job control and demands scores ranged from 0 to 10, with the higher scores indicating higher levels. The median scores of job control (6.8 for men; 5.8 for women) and job demands (6.1 for men; 6.2 for women) in the matrix were used to dichotomize these two dimensions. Further, we generated four demand-control categories: high strain (low control and high demands), low strain (high control and low demands), passive job (low control and low demands), and active job (high control and high demands).

### Diagnosis of dementia

Dementia is a broad term used to describe different conditions affecting the brain, and there are a number of subtypes. In the current study, we focused on dementia of any type, including neurodegenerative and vascular forms. At baseline and all follow-ups, dementia was diagnosed following the Diagnostic and Statistical Manual of Mental Disorders, 4^th^ Edition criteria. Following a validated three-step procedure, the first physician made the preliminary diagnosis based on interviews, clinical examinations, and cognitive testing, and then a reviewing physician made the second diagnosis. In case of disagreement, a third opinion from a senior neurologist was sought to reach a concordant diagnosis. For those who died before the subsequent follow-up examination, incident dementia was diagnosed by physicians through an extensive review of medical records, discharge diagnoses, and death certificates.

### Covariates

The highest degree of education achieved was recorded as elementary, high school, and university. According to a previous report [33], current engagement in leisure activities (including mental, social and physical) was categorized into three levels as low, moderate, and high. Early-life SES was assessed using occupation of participant’s father and categorized into low, intermediate, and high. *APOE* genotypes were identified using a microsequencing method (AffiGen *APOE*, Sangtec Medical) based on a polymerase chain reaction with biotinylated primers. *APOE* allelic status was dichotomized as any ɛ4 carriers and ɛ4 non-carriers. Heart diseases, including atrial fibrillation, ischemic heart disease, heart failure, cardiac valve disease, bradycardias and conduction diseases, were ascertained by means of clinical examinations, medical history or current drug use, and the link to the Swedish National Patient Register.

### Statistical analysis

Baseline characteristics between participants with different demand-control categories were compared using Chi-square (χ^2^) or one-way ANOVA followed by pairwise comparison with Bonferroni correction. We also examined differences between incident dementia cases and non-cases.

In accordance with previous studies, job demands and control in the longest-held job were chosen to be the primary exposures. Cox proportional hazards regression models were used to estimate the hazard ratios (HRs) and 95% confidence intervals (95% CIs) of dementia in relation to job control, job demands, and demand-control combinations. Follow-up time was calculated from the date of baseline examination until date of death or date of last examination for dementia-free individuals. For incident cases, the date of dementia diagnosis was either the date of examination or the date of death. The proportional hazard assumption was tested by regressing the scaled Schoenfeld’s residuals against survival time. No violation of proportionality was detected.

Statistical interactions between demand-control status and *APOE* genotype, as well as sex, education, and early-life SES, with respect to estimating dementia risk, were tested by introducing interaction terms in the model. To test the joint effect of occupational status and *APOE* ɛ4 on dementia, we created an eight-category indicator variable by cross-tabulating four job categories (i.e., high strain, low strain, passive job, or active job) and two *APOE* genotypes (i.e., presence or absence of ɛ4).

Further, to assess the association of cumulative exposure to demand-control conditions with dementia, we calculated total years spent in each demand-control category based on the duration of the five jobs (i.e., the most recent job and four longest-held jobs). For each job category, duration of work was categorized into zero years, less than or equal to median years of work, or greater than median years of work.

Stratified analyses were carried out by two age cohorts, the younger-old (aged ≤72 years) and the older-old (aged ≥78 years), for several reasons. First, the younger-old and older-old individuals are generally at different degrees of dementia risk because of age [2]. Second, the work exposure was more recent among the younger-old than the older-old, while older-old dementia cases have higher heterogeneity which could result from a more complex combination of risk factors and chronic conditions [27]. Thus, it is plausible to hypothesize that the relationship between working conditions and dementia may differ between the two age groups. Third, we previously reported the association of demand-control status with dementia in the KP population, which consisted of people aged 75+ years living in the same geographical area of the SNAC-K population twenty years ago. We pondered whether the association between working conditions and dementia in the older-old has changed considering the ongoing changes in education, labor market and awareness of disease prevention in the Western societies [19–24]. Comparing the job-dementia relationship between the older-old in SNAC-K and participants in KP might help examine the cohort effects on this association.

All the analyses were first adjusted for age, sex and education, and additionally controlled for heart diseases, leisure activity engagement, and early-life SES.

Several sensitivity analyses were carried out. First, we excluded individuals who had an MMSE score lower than 24 at baseline or who were diagnosed with dementia during the first three years of follow-up (n=166). Then, we repeated the analyses where baseline MMSE score was additionally controlled for. This was to minimise information bias due to poor cognition, as well as to take into consideration the potential reverse causality. Second, people aged 60 years and working at baseline (n=464) were excluded to eliminate the potential impact of current job on dementia. Third, since the majority of people retired at the age of 65, participants who took early retirement (i.e., retired before the age of 65) due to disability, life plan, or other reasons (n=25) were excluded. This was done because their characteristics are more heterogeneous and less generalizable to the majority of older adults in Sweden. Fourth, we excluded those diagnosed with dementia after death (n=67) to eliminate diagnostic bias. Fifth, we also assessed working conditions using the most recent job to reduce potential recall bias. Finally, we performed multiple imputation by chained equations for missing data on covariates, obtaining five imputed datasets. The estimates from these datasets were pooled according to Rubin's rule for valid statistical inferences. All analyses were computed using Stata SE 15.0 (StataCorp LP., College Station, TX).

## Supplementary Material

Supplementary Figures

Supplementary Tables
