# Supplementary material for: The role of Apolipoprotein E epsilon4 in the association between psychosocial working conditions and dementia
Source: Aging (Albany NY). 2020 Feb 20;12(4):3730–46. doi: 10.18632/aging.102843 (PMC7066897; doi:10.18632/aging.102843)
Supplement: Supplementary Figures [file aging-12-102843-s001..pdf]

SUPPLEMENTARY FIGURES

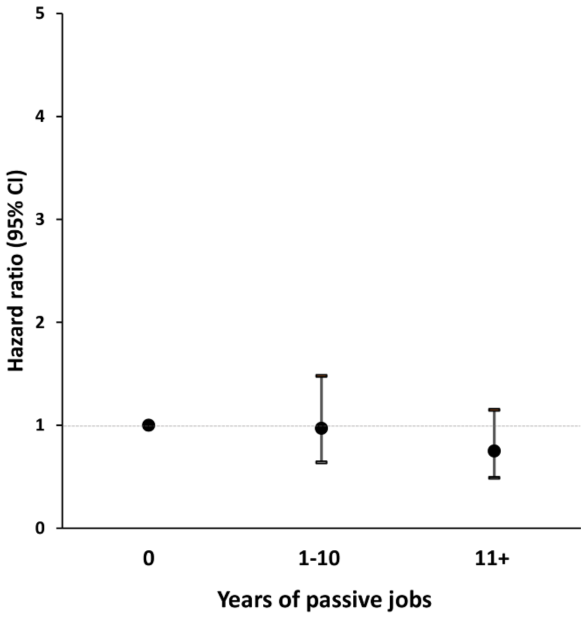

No. subjects	735	153	175
No. cases	159	31	29

Supplementary Figure 1. Hazard ratios (HRs) and 95% confidence intervals (CIs) of incident dementia associated with duration of passive jobs among adults aged ≥78 years. Cox regression model was adjusted for age, sex, education, heart diseases, leisure activity engagement, and early-life socioeconomic status.

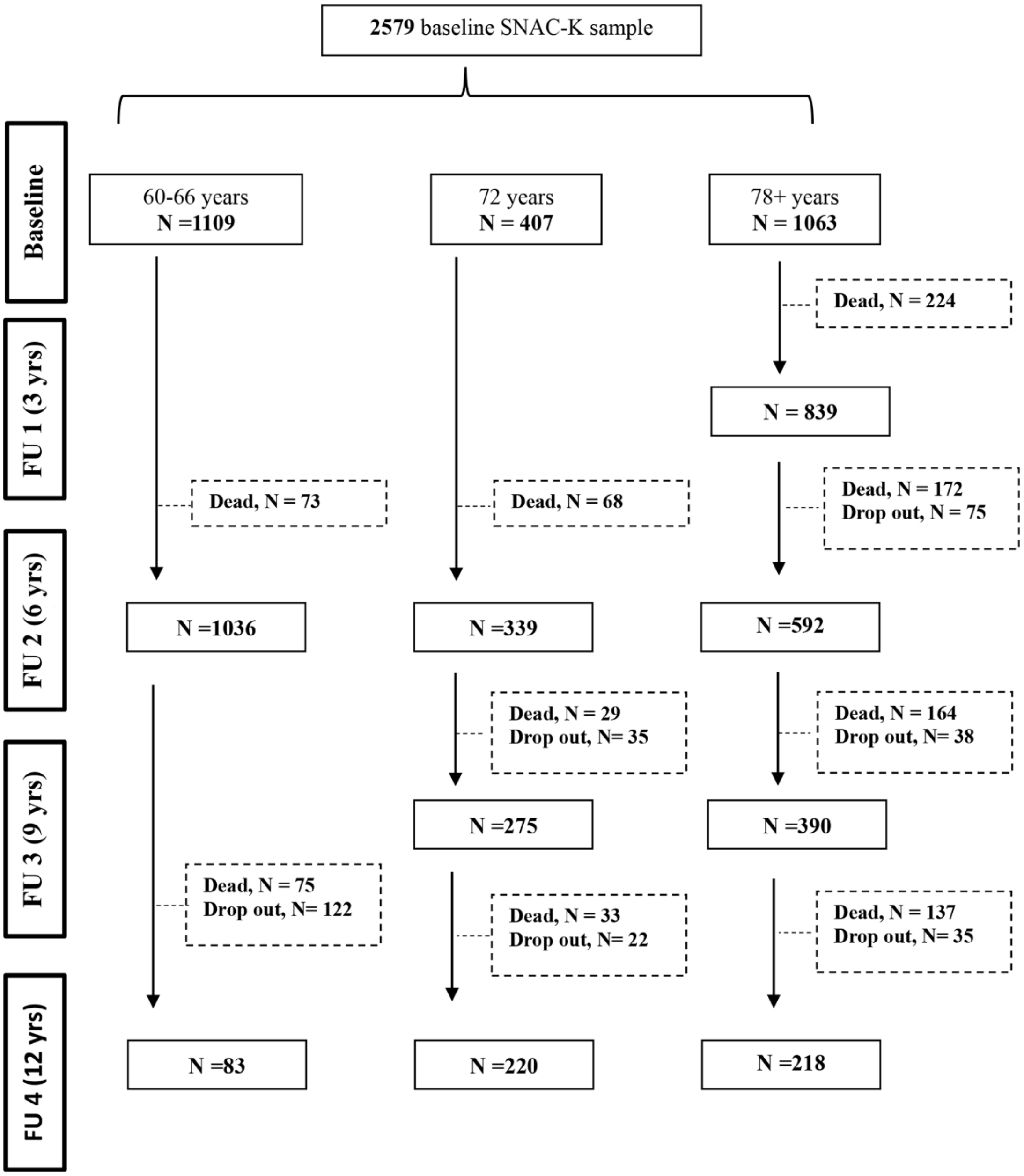

Supplementary Figure 2. Flow chart of study participation over 12 years. Dropouts were due to either refusal of the participant/relative, loss of contact with the participant, or moving of the participant from the city where the study took place.
