# Supplementary material for: The role of Apolipoprotein E epsilon4 in the association between psychosocial working conditions and dementia
Source: Aging (Albany NY). 2020 Feb 20;12(4):3730–46. doi: 10.18632/aging.102843 (PMC7066897; doi:10.18632/aging.102843)
Supplement: Supplementary Tables [file aging-12-102843-s002..pdf]

SUPPLEMENTARY TABLES

Supplementary Table 1. Characteristics of people aged ≤72 years by demand-control status of the longest-held job.

**Characteristics**	**Active**	**Low Strain**	**High Strain**	**Passive**	***p***
	**n=1064**	**n= 196**	**n=158**	**n=98**	
Age (years)	65 ± 5	66 ± 5	66 ± 5	65 ± 5	<0.01
Female sex	580 (54.5)	136 (69.4)	103 (65.2)	49 (50.0)	<0.001
Education					
Elementary	50 (4.7)	23 (11.7)	17 (10.8)	32 (32.7)	
High school	419 (39.4)	129 (65.8)	91 (57.6)	54 (55.1)	<0.001
University	595 (55.9)	44 (22.5)	50 (31.6)	12 (12.2)	
Leisure activity engagement					
Low	199 (19.9)	48 (26.8)	26 (18.2)	34 (38.2)	
Moderate	472 (47.1)	92 (51.4)	70 (48.9)	36 (40.5)	<0.001
High	331 (33.0)	39 (21.8)	47 (32.9)	19 (21.3)	
Early-life socioeconomic status					
Low	333 (32.7)	81 (43.3)	73 (48.4)	48 (52.2)	
Intermediate	341 (33.5)	52 (27.8)	39 (25.8)	24 (26.1)	<0.001
High	345 (33.8)	54 (28.9)	39 (25.8)	20 (21.7)	
Heart diseases	113 (10.6)	29 (14.8)	17 (10.8)	19 (19.4)	<0.05
APOE ε4 carriers	315 (30.7)	52 (27.5)	50 (33.1)	31 (33.3)	0.66
MMSE score	29.3 ± 0.9	29.1 ± 1.1	29.2 ± 1.0	28.7 ± 1.5	<0.001
12-year follow-up					
Incident dementia	40 (3.8)	10 (5.1)	4 (2.5)	9 (9.2)	<0.05

Data are presented as mean ± standard deviations or number (proportion %).
Abbreviations: APOE ε4, apolipoprotein ε4 allele; MMSE, Mini-Mental State Examination.

Supplementary Table 2. Characteristics of people aged ≥78 years by demand-control status of the longest-held job.

Characteristics	Active	Low Strain	High Strain	Passive	*p*
	n=547	n= 225	n=134	n=157	
Age (years)	83 ± 5	84 ± 6	84 ± 6	85 ± 6	<0.001
Female sex	335 (61.2)	180 (80.0)	96 (71.6)	123 (78.3)	<0.001
Education					
Elementary	57 (10.4)	61 (27.1)	44 (32.8)	93 (59.2)	
High school	306 (55.9)	147 (65.3)	81 (60.5)	59 (37.6)	<0.001
University	184 (33.7)	17 (7.6)	9 (6.7)	5 (3.2)	
Leisure activity engagement					
Low	177 (39.2)	88 (50.3)	42 (43.8)	51 (45.1)	
Moderate	189 (41.8)	74 (42.3)	42 (43.8)	54 (47.8)	0.001
High	86 (19.0)	13 (7.4)	12 (12.4)	8 (7.1)	
Early-life socioeconomic status					
Low	180 (34.1)	106 (50.5)	64 (50.8)	93 (62.8)	
Intermediate	165 (31.2)	57 (27.1)	30 (23.8)	38 (25.7)	<0.001
High	183 (34.7)	47 (22.4)	32 (25.4)	17 (11.5)	
Heart diseases	195 (35.7)	89 (39.6)	44 (32.8)	64 (40.8)	0.39
APOE ε4 carriers	135 (26.5)	58 (28.7)	28 (22.8)	36 (25.2)	0.68
MMSE score	28.3 ± 1.7	27.8 ± 2.1	27.9 ± 1.9	27.4 ± 2.3	<0.001
12-year follow-up					
Incident dementia	143 (26.1)	63 (28.0)	44 (32.8)	44 (28.0)	0.49

Data are presented as mean ± standard deviations or number (proportion %).
Abbreviations: APOE ε4, apolipoprotein ε4 allele; MMSE, Mini-Mental State Examination.

Supplementary Table 3. Characteristics of incident dementia cases and non-cases stratified by age.

Characteristics	Aged ≤72		p	Aged ≥78		p
	Non-case n=1453	Case n=63		Non-case n=844	Case n=219	
Age (years)	65 ± 5	71 ± 3	<0.001	83 ± 5	84 ± 5	0.26
Female sex	834 (57.4)	34 (54.0)	0.59	578 (68.5)	156 (71.2)	0.43
Education						
Elementary	115 (7.9)	7 (11.1)		213 (25.2)	42 (19.2)	
High school	655 (45.1)	38 (60.3)	<0.05	452 (53.6)	141 (64.4)	<0.05
University	683 (47.0)	18 (28.6)		179 (21.2)	36 (16.4)	
Leisure activity engagement						
Low	289 (21.2)	18 (34.6)		276 (40.4)	82 (53.6)	
Moderate	646 (47.5)	24 (46.2)	<0.05	303 (44.4)	56 (36.6)	<0.01
High	426 (31.3)	10 (19.2)		104 (15.2)	15 (9.8)	
Early-life socioeconomic status						
Low	505 (36.3)	30 (50.8)		350 (43.5)	93 (44.7)	
Intermediate	446 (32.1)	10 (17.0)	<0.05	230 (28.6)	60 (28.9)	0.92
High	439 (31.6)	19 (32.2)		224 (27.9)	55 (26.4)	
Heart diseases	178 (12.3)	16 (25.4)	<0.01	331 (39.2)	88 (40.2)	0.80
APOE ε4 carriers	421 (30.0)	27 (46.6)	<0.01	189 (24.2)	68 (34.7)	<0.01
MMSE score	29.3 ± 0.9	28.2 ± 1.7	<0.001	28.2 ± 1.9	27.4 ± 2.0	<0.001
Demand-control category						
Active	1024 (70.5)	40 (63.5)	<0.05	436 (51.7)	111 (50.7)	0.65
Low strain	186 (12.8)	10 (15.9)		180 (21.3)	45 (20.6)	
High strain	154 (10.6)	4 (6.3)		101 (12.0)	33 (15.1)	
Passive	89 (6.1)	9 (14.3)		127 (15.1)	30 (13.7)	

Data are presented as mean ± standard deviations or number (proportion %).
Abbreviations: *APOE* ε4, apolipoprotein ε4 allele; MMSE, Mini-Mental State Examination.

Supplementary Table 4. Hazard ratios (HRs) and 95% confidence intervals (CIs) of incident dementia associated with job control and job demands in the total sample and in two age cohorts.

Job control<br>Job demands	N	No. of cases	Model 1[a]		Model 2[b]	
			HR (95% CI)	*p*	HR (95% CI)	*p*
**Overall**						
High control	2032	206	Ref.		Ref.	
Low control	547	76	0.99 (0.75-1.32)	0.95	0.99 (0.74-1.33)	0.95
High demands	1903	188	Ref.		Ref.	
Low demands	676	94	1.05 (0.81-1.38)	0.68	1.04 (0.79-1.37)	0.81
**Aged ≤72**						
High control	1260	50	Ref.		Ref.	
Low control	256	13	1.06 (0.56-2.02)	0.85	1.07 (0.55-2.07)	0.84
High demands	1222	44	Ref.		Ref.	
Low demands	294	19	1.37 (0.76-2.45)	0.29	1.30 (0.71-2.39)	0.40
**Aged ≥78**						
High control	772	156	Ref.		Ref.	
Low control	291	63	1.04 (0.76-1.43)	0.80	1.02 (0.74-1.42)	0.89
High demands	681	144	Ref.		Ref.	
Low demands	382	75	0.97 (0.72-1.30)	0.82	0.96 (0.70-1.31)	0.80

[a] Adjusted for age, sex, and education; [b] Adjusted for age, sex, education, heart diseases, leisure activity engagement, and early-life socioeconomic status.
